# Gamma Index Analysis as a Patient-Specific Quality Assurance Tool for High-Precision Radiotherapy: A Clinical Perspective of Single Institute Experience

**DOI:** 10.7759/cureus.30885

**Published:** 2022-10-30

**Authors:** Saikat Das, Vipin Kharade, VP Pandey, Anju KV, Rajesh K Pasricha, Manish Gupta

**Affiliations:** 1 Department of Radiation Oncology, All India Institute of Medical Sciences, Bhopal, IND

**Keywords:** radiotherapy phantom, quality control in radiotherapy, volumetric-modulated arc therapy, intensity modulated radiotherapy, gamma index

## Abstract

Purpose

Patient-specific quality assurance (QA) by gamma (γ) analysis is an important component of high-precision radiotherapy. It is important to standardize institute-specific protocol. In this study, we describe our institutional experience of patient-specific QA for high-precision radiotherapy from a clinical perspective.

Methods

The planning data of 56 patients treated with intensity-modulated radiotherapy (IMRT)/volumetric modulated arc therapy (VMAT) were included. γ index analysis was done using Octavius 4D IMRT QA phantom (PTW, Freiburg, Germany) using 3 mm/3% criteria. Local, global, and volumetric gammas were calculated and compared. The relationship of γ index in the transverse, coronal, and sagittal direction and anatomical region of treatment was explored.

Results

Global three-dimensional (3D) γ indices in the coronal, sagittal, and transverse axes were 96.73 ± 2.35, 95.66 ± 3.01, and 93.36 ± 4.87 (p < 0.05). The average local two-dimensional (2D) γ index was 78.23 ± 5.44 and the global γ index was 92.41 ± 2.41 (p < 0.005). The average local 3D γ index was 84.99 ± 4.24 and the global 3D γ index was 95.25 ± 1.72 (p < 0.005, paired t-test). The average local volumetric γ index was 84.29 ± 4.73 and the global volumetric γ index was 95.96 ± 2.08 (p < 0.005). 3D global gamma index was significantly different in different anatomical regions (p < 0.05).

Conclusion

Our study shows that γ index analysis is a useful parameter for routine clinical IMRT QA. The choice of type of γ index depends on the context of use and degree of stringency in measurement. Average 2D and 3D global γ were different in anatomical regions. The average 3D γ index was significantly different in axes. No difference was observed with techniques of IMRT/VMAT. Localization of failed points in CT anatomy can be advantageous for clinical decision-making.

## Introduction

High-precision radiotherapy techniques like intensity-modulated radiotherapy (IMRT) and volumetric modulated arc therapy (VMAT) can improve the therapeutic ratio through the escalation of tumor dose and superior target coverage simultaneously sparing the normal tissue [1]. In VMAT, volumetric modulation can be achieved by varying several parameters like multileaf collimator's (MLC) aperture shape, the fluence-output rate, and the gantry rotation speed [2].

The movement of MLC produces complex dose distribution and intensity modulation in the IMRT technique. There is a considerable increase in the possibility of dosimetric variation due to the inherent complexity of these treatment techniques. Therefore, a three-dimensional (3D) dose matrix, with a volumetric evaluation of composite fields, is superior to a planar dose value map [3]. The difference between the treatment planning system (TPS) and the verification plan, relative to the accepted tolerance, is indicated by a quality measurement called the “Gamma (γ) index” [4]. This is very useful in the quantification and comparison of two-dimensional (2D) and 3D dose data, where direct measurement remains challenging. The dose gradient factor represents a relative allowance between spatial and dosimetric shift, and “pass-fail” criteria of both dose difference (DD) and distance to agreement (DTA) have been reported in the literature based on selected tolerance levels [5].

Current clinical standards of 3% DD criterion (ΔDM) and 3 mm DTA criterion (ΔdM) are widely considered acceptable for photon beams [4]. A 90% passing rate with 3 mm/3% gamma analysis as suggested by TG 119 is followed in many institutions [6]. The American Association of Physicists in Medicine (AAPM) TG 218 report provides a comprehensive explanatory guideline for the use of the gamma index for patient-specific IMRT quality assurance (QA) [7]. Various commercially available dosimetric verification systems for gamma analysis have been reported in the literature. Octavius 4D phantom (PTW, Freiburg, Germany) and VeriSoft software (PTW, Freiburg, Germany) used in IMRT/VMAT QA process have been evaluated for high-precision radiotherapy treatments including stereotactic ablative radiotherapy (SABR) [3,8]. Since γ pass rate varies according to the type of γ analysis, dosimeter, TPS, and linear accelerator setup [9], it is important to standardize institute-specific protocol including the type of gamma metric to be used. This study describes our institutional experience of patient-specific QA for high-precision radiotherapy. We explored the relationship between different γ index metrics and their correlation with different anatomical regions of treatment and directional axes.

## Materials and methods

The planning data of 56 patients treated with IMRT/VMAT were included. The study was approved by Institutional Human Ethics Committee, AIIMS Bhopal (IHEC-LOP/2021/ IM0343).

Octavius 4D IMRT phantom commissioning

The commissioning of Octavius 4D was carried out with the Elekta Versa HD medical linear accelerator (Elekta, Stockholm, Sweden). Percentage depth dose (PDD) was measured for field sizes ranging from 4 × 4 cm^2^ to 26 × 26 cm^2^ measured at 85 cm source to surface distance (SSD). The Octavius 4D CT used for plan verification was the artificial one provided by the vendor. However, to better model the phantom in the TPS, a CT scan of the phantom was performed in Simulator CT (Optima CT 580, Wipro GE Healthcare, Singapore) and the obtained averaged Hounsfield unit (HU) was reported into the TPS. Moreover, the correct distance from the couch was measured and reported in the artificial CT by fusion of images. Static delivery tests included 5 × 5, 10 × 10, 20 × 20 cm^2^ static fields and a “pyramid” field‐in‐field shape given by superimposition of 5 × 5, 10 × 10, and 20 × 20 cm^2^ static fields.

The evaluation of arc delivery performance was obtained using a 5 × 5 cm^2^ arc and the combination of 5 × 5 and 10 × 10 cm^2^ arcs, with a clockwise 180° rotation for both arcs, which were sequentially delivered to cover a full rotation of the phantom. The effect of different spatial directions was assessed by considering transversal, coronal, and sagittal planes and verifying whether there was a different response or not. Dosimetric data were assessed by the γ analysis with acceptance criteria of 3%/3 mm. To achieve absolute dose, the central chamber of the array was cross‐calibrated with a PTW semiflex ionization chamber (volume: 0.125 cc, type 31010), inserted into an RW3 slab replacing the 2D array inside Octavius 4D. The position of the chamber with respect to the isocenter was preliminarily verified by orthogonal kV images obtained in Elekta Versa HD On‐Board Imager (OBI).

Measurements at the reference condition were carried out (field 10 × 10 cm^2^, gantry at 0°, 200 MU corresponding to 1.248 Gy for Versa HD, i.e., the expected value in each measure session for the clinical plan verification) and the dose value was deduced from the chamber signal following the International Atomic Energy Agency (IAEA) Technical Report Series 398 approach, taking into account also the correction for the daily linear accelerator (LINAC) output factor. The comparison with the central chamber measurement at the same conditions allowed deducing the so‐called Kuser factor for each LINAC, useful for validation of Octavius 4D for absolute dose assessment. This result was compared with the Kcross, to evaluate the consistency of the two approaches. Finally, it was evaluated that the dose distribution obtained by two uninterrupted clinical arc deliveries vs. the same arcs delivered with up to four interruptions to understand the performance of the LINAC when an undesirable arc interruption happens and to test the inclinometer reliability.

Patient-specific quality assurance

The treatment QA delivery was performed in Elekta Versa HD LINAC (Elekta Instrument AB, Stockholm, Sweden) with a 6 MV beam for all fields. The γ index metric was computed using Octavius 4D phantom and VeriSoft software. As a detector, the PTW Octavius Detector 1500 array was used, which has a high resolution (0.1 mGy) with 1405 chambers arranged as a checkboard of size 4.4 × 4.4 × 3 mm (0.06 cm³) in 27 × 27 cm area. The inclinometer setup allowed the phantom to be synchronized with the rotation speed and angle of the gantry of the linear accelerator as in actual treatment delivery. The direction of the beam always remains perpendicular to the detector array, avoiding any additional correction factor for beam direction.

The dose distribution of the treatment plan was recalculated on the CT of the phantom performed in the simulator CT to generate the verification plan. The dosimetric verification was carried out by comparing the measured plan with the verification plan. Cross calibration based on the reference value provided by the TPS defrayed the output variations in LINAC. Dosimetric information was independent of TPS due to the application of the correction factor to the whole detector matrix and the conversion of PDD from water to phantom material. The 2D and 3D dosimetric metric called the “γ index” in different axes (local, global, and volumetric) were obtained by processing the data in VeriSoft software.

Gamma analysis

The 2D, 3D, and volumetric γ were evaluated with a 3%/3 mm acceptance criterion [3]. The 3%/3 mm acceptance criterion was selected as it is widely used as a clinical standard [4]. Results by both local and global γ analysis definitions were investigated. The dose difference in γ analysis was increased to 5% for doses lower than 0.1 Gy with respect to the maximum dose as reported in the literature [10]. The γ index in all three axes (transverse, coronal, and sagittal) was calculated (2D/3D, global, and local) in addition to the volumetric γ index.

Statistical analysis

Statistical analysis was performed with IBM SPSS Statistics software (IBM Corp., Armonk, NY). Gaussian distribution of data was tested for normalcy by the Kolmogorov-Smirnov test. A parametric test (t-test) was used for the comparison of the mean and analysis of variance (ANOVA) was studied for the comparison of different groups. For statistical analysis, the significance level was taken as p < 0.05.

## Results

Octavius 4D commission

**Figure 1:**
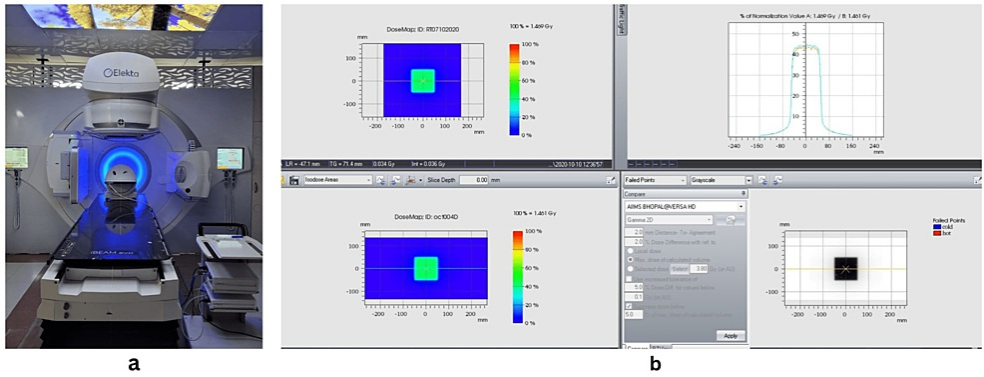
IMRT phantom and commissioning Left: (a) Octavius 4D IMRT phantom. (b) Right: Octavius commissioning for 10 x 10 field size showing the comparison of planned and measured dose. The figure shows the Octavius phantom setup in LINAC and the dose profile of the static 10 x 10 cm^2^ field. IMRT: intensity-modulated radiotherapy; LINAC: linear accelerator.

The low dose threshold for the inconsistent area of comparison was set at 0.1% of the maximum dose and the cut-off threshold was set at 5%. The Octavius phantom set up in LINAC and the dose profile of the static 10 × 10 cm^2^ field are shown in Figure 1.

Patient, tumor, and treatment-related factors

The mean age of the cohort was 48 years (23-78 years). The mean PTV volume was 496.65 cc (230.34-1065.96). Treatment technique involved IMRT in 11 cases and VMAT in 45 cases. Radiotherapy treatment involved the cranial region (n = 28), thoracic region (n = 14), and pelvic region (n = 14).

Comparison between different types of gamma metric

Local 2D γ indices in coronal, sagittal, and transverse axes were 82.48 ± 6.29, 80.12 ± 6.02, and 72.1 ± 9.4, and global 2D γ indices in the respective axes were 94.42 ± 3.63, 93.08 ± 4.05, and 89.74 ± 6.74.

Local 3D γ indices in coronal, sagittal, and transverse axes were 88.43 ± 5.26, 86.3 ± 5.14, and 80.25 ± 8.37, and global 3D γ indices in the respective axes were 96.73 ± 2.35, 95.66 ± 3.01, and 93.36 ± 4.87. The average (across three axes) 2D local γ index was 78.23 ± 5.44 and the global γ index was 92.41 ± 2.41 (p < 0.005, paired t-test). The average 3D local gamma index was 84.99 ± 4.24 and the global 3D gamma index was 95.25 ± 1.72 (p < 0.005, paired t-test; Table 1).

**Table 1:** Mean and standard deviation of 2D, 3D, and volumetric gamma index (local and global) 2D: two-dimensional; 3D: three-dimensional.

	2D	3D	Volumetric
	Average on three axes	Average on three axes	
Local	78.23 ± 5.45	84.99 ± 4.24	84.29 ± 4.73
Global	92.41 ± 2.41	95.25 ± 1.72	95.96 ± 2.08

**Figure 2:**
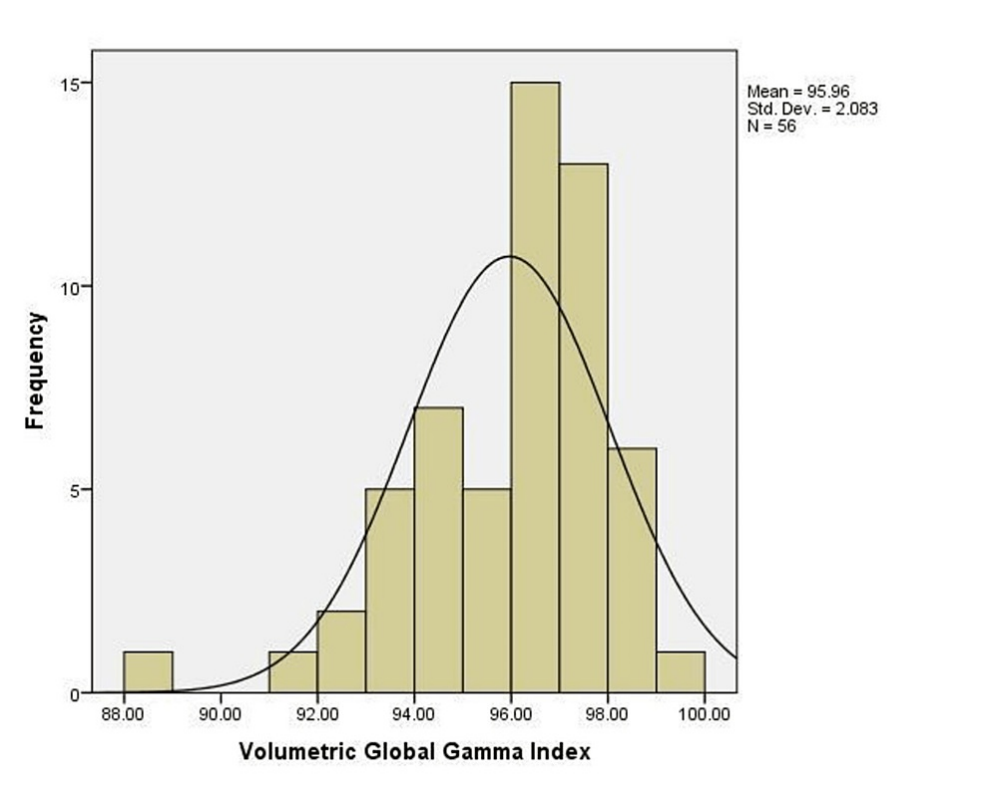
Histogram showing volumetric global gamma index. The graph shows the normal distribution of the volumetric gamma index with a mean of 95.96 ± 2.08

**Figure 3:**
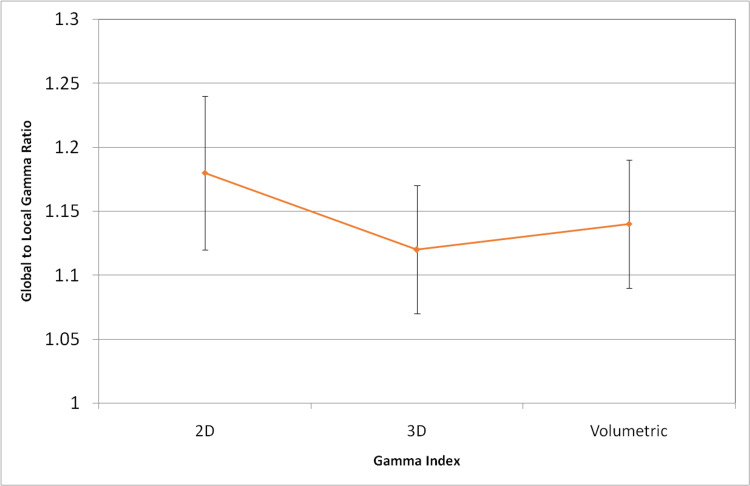
Graph showing global to local gamma ratio Y axis = global to local gamma ratio; X axis = 2D, 3D, and volumetric gamma. The ratio of global gamma index to local gamma index (Y-axis) was lower for 3D (1.12 ± 0.05) and volumetric gamma (1.14 ± 0.05) than 2D gamma (1.18 ± 0.06) (p < 0.05). 2D: two-dimensional; 3D: three-dimensional.

The average local volumetric γ index was 84.29 ±4.73 and the global volumetric γ index was 95.96 ± 2.08 (p < 0.005). It was normally distributed (Figure 2). In 55 cases, a percentage of at least 90% passed points for the global volumetric gamma index was observed. The mean global γ index was 95.89 ± 2.50 for IMRT (n = 11) and 95.97 ± 1.99 for VMAT (n = 45, p = NS). The ratio of global gamma index to local gamma index was lower for 3D (1.12 ± 0.05) and volumetric gamma (1.14 ± 0.05) than 2D gamma (1.18 ± 0.06, p < 0.05, Figure 3).

Local and global gamma indices were significantly correlated. Pearson correlation coefficients for 2D, 3D, and volumetric local and 2D global gammas were statistically significant (p < 0.05; r = 0.714, 0.624, and 0.563, respectively).

Directional dependence of γ index

**Figure 4:**
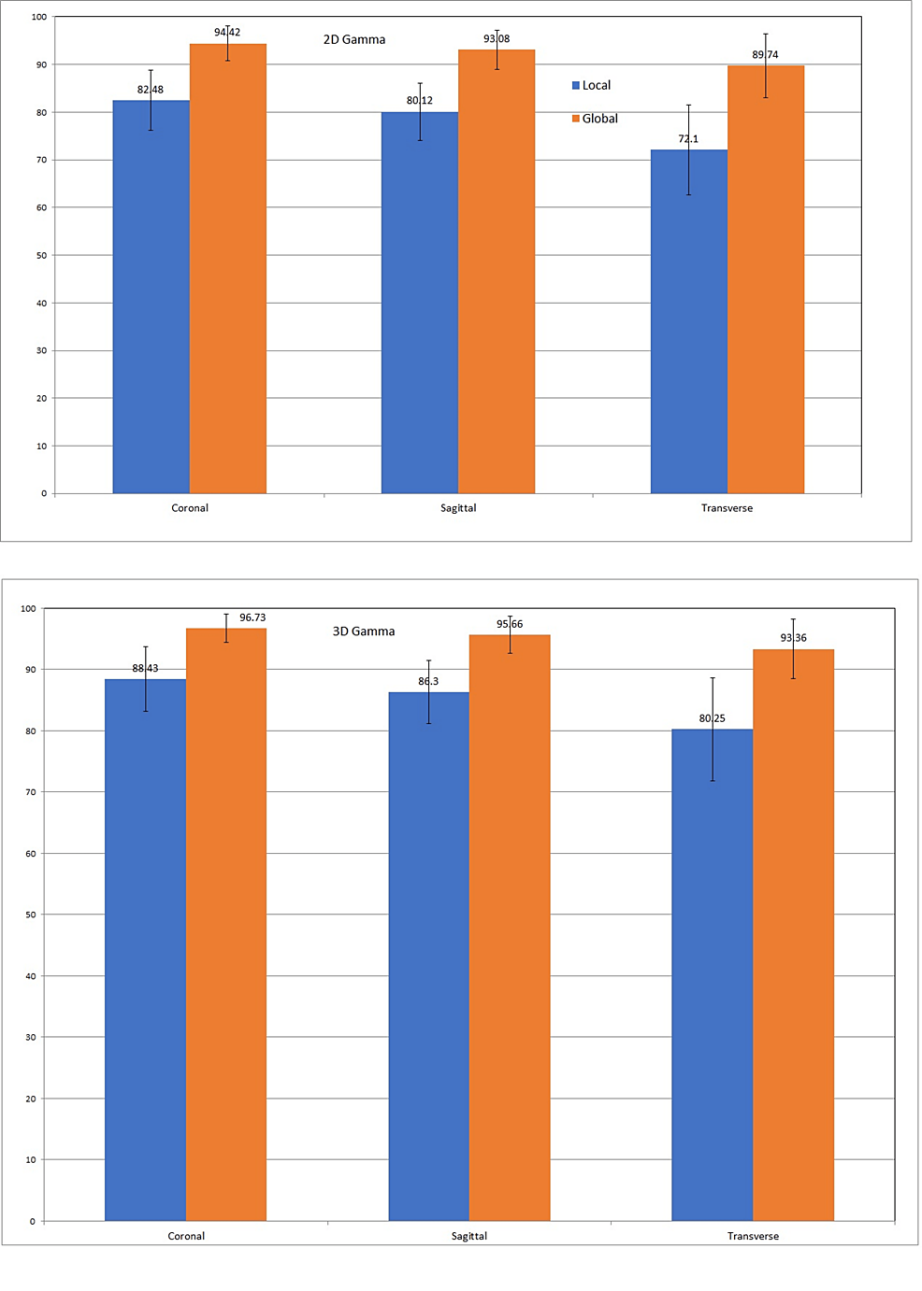
The directional dependence of gamma index to coronal, sagittal, and transverse directions. The upper panel shows the 2D gamma index and the lower panel shows the 3D gamma index 2D: two-dimensional; 3D: three-dimensional.

The result of concordance of γ index depending on the section of the phantom in three different axes (coronal, sagittal, and transverse) is shown in Figure 4. The average 2D and 3D global γ index were significantly different among the coronal, sagittal, and transverse axes (p < 0.005, ANOVA test) (Table 2).

**Table 2:** Directional dependence of gamma index on axes of measurement 2D: two-dimensional; 3D: three-dimensional.

Gamma index	Direction	Mean	Std. error	P-value
2D local	Coronal	82.47	0.84	<0.005
	Sagittal	80.12	0.80	
	Transverse	72.10	1.25	
2D global	Coronal	94.42	0.48	<0.005
	Sagittal	93.08	0.54	
	Transverse	89.74	0.90	
3D local	Coronal	88.43	0.70	<0.005
	Sagittal	80.12	0.80	
	Transverse	72.10	1.25	
3D global	Coronal	96.73	0.31	<0.005
	Sagittal	95.66	0.40	
	Transverse	93.36	0.65	

Relationship of γ index and anatomical region of treatment

The average 2D global γ indices in the cranial region (n = 28), thoracic region (n = 14), and pelvic region (n = 14) were 91.85, 94.85, and 94.85, respectively (p < 0.05, ANOVA test). The average 3D global γ indices in the cranial region (n = 28), thoracic region (n = 14), and pelvic region (n = 14) were 94.68, 97.08, and 94.56, respectively (p < 0.05, ANOVA test). There was no statistically significant difference in global or local volumetric γ index across the regions (Table 3).

**Table 3:** Dependence of gamma index and anatomical region of treatment * = statistically significant (p < 0.05). 2D: two-dimensional; 3D: three-dimensional.

Gamma index	Region of radiotherapy		Mean	Std. error	P-value
Average 2D local (average over all axes)		Cranial	77.67	0.875	0.46
		Thoracic	79.86	2.08	
		Pelvic	77.72	1.31	
Average 2D global (average over all axes)		Cranial	91.85	0.78	0.01*
		Thoracic	94.85	0.74	
		Pelvic	91.11	0.91	
Average 3D local (average over all axes)		Cranial	84.43	0.68	0.60
		Thoracic	86.08	1.78	
		Pelvic	85.05	1.48	
Average 3D global (average over all axes)		Cranial	94.68	0.58	0.02*
		Thoracic	97.08	0.49	
		Pelvic	94.56	0.758	
Volume local		Cranial	83.54	0.62	0.48
		Thoracic	85.26	1.69	
		Pelvic	84.83	1.45	
Volume global		Cranial	95.52	0.41	0.06
		Thoracic	97.06	0.45	
		Pelvic	95.72	0.51	

## Discussion

The gamma index is a useful tool for dosimetric verification that compares the TPS plan to the measured plan and provides the metric of the agreement to dose. It is widely used as a patient-specific IMRT QA [9,11]. It is important to develop an institute-specific protocol as the gamma index depends on the planning and treatment setup. The type of dosimeter and resolution of the detector, TPS algorithm, linear accelerator setup, and clinical judgment of dose tolerance level also influence the result [8,9]. In this study, we explored the relationship between different categories of the gamma index. The global gamma index is calculated with respect to the maximum dose and produces more homogeneous results with a higher passing rate [3]. The local gamma index is calculated with respect to the reference point. 2D gamma index calculation considers each slice as a separate plane irrespective of surrounding volume. In 3D analysis, slice-by-slice agreement taking into account neighboring planes is calculated. Therefore, for the same cut-off value, 3D gamma results in more passing rate than 2D gamma.

Volumetric γ evaluation assesses the entire volume considering that it is exposed to the radiation in a time‐resolved mode [3]. Although 3D and volumetric gamma are a natural extension of the 2D gamma concept, the clinical significance of these metrics in patient-specific QA requires further studies [11,12]. One advantage of volumetric analysis is that the user can identify the dose error away from the central axis plane, which is very useful in cases where a small volume is encompassed with a high dose gradient [12]. It is noteworthy that the DTA and DD criteria used for gamma analysis are not completely independent. They are related to the dose gradient factor. Clinically 3 mm/3% for a 90% passing rate is widely accepted and the detection limit has been reported to be 4.07 mm/4.07% [5]. The global or local gamma index should be used in the context of use. Global normalization is clinically more relevant than local normalization in patient treatment whereas more stringent local normalization can be useful for IMRT commissioning and troubleshooting QA [7].

Octavius 4D phantom is a useful tool for patient-specific QA in IMRT/VMAT [2,3,11]. Our study confirms that the single slice evaluation (2D) had lesser agreement compared to 3D and volumetric γ‐index (p < 0.001). The same result was found by Urso et al. [3]. In a study by Stathakis et al., 3D gamma measurements varied from 92.3% to 99.9% [11]. Our study shows the average 3D global γ (95.25, across axes) is almost similar to the volumetric gamma index (95.96, p = NS). Our findings are consistent with the literature [3]. Our study shows that 2D and 3D local and global gammas were significantly different in three directions. Average 2D and 3D global gammas were different in anatomical regions. Local and global volumetric gammas were significantly different but correlated. No difference was observed with techniques or with anatomical regions.

In a previous study, the average volumetric 3D global gamma indices (for head and neck, pelvic, and thoracic regions) for VMRT plans were reported to be 95.45%, 97.51%, and 96.91% [2]. Our study is consistent with that reported in the literature (corresponding values in the present study were 95.52%, 95.72%, and 97.06%). Though there are differences in planning techniques, it may be mentioned that the modulation complexity score (MCS) of a plan has a weak correlation with local or global gamma analysis passing rate [2,9]. MCS is a measure of plan complexity in VMAT [13] and further studies are required to explore the relationship between gamma index and beam complexity including monitor units [14].

Park et al. reported a weak correlation between local and global gamma index at the 3 mm/3% cut-off level (Pearson correlation coefficient r = 0.218, p = NS, ArcCHECK dosimeter). In our study, we found a significant correlation between the local and global gamma index. This could be because the dosimetric verification system and resolution of detectors were different. Gamma analysis varies depending on the type of dosimeter used and variation between different dosimeters could be considerable [9].

**Figure 5:**
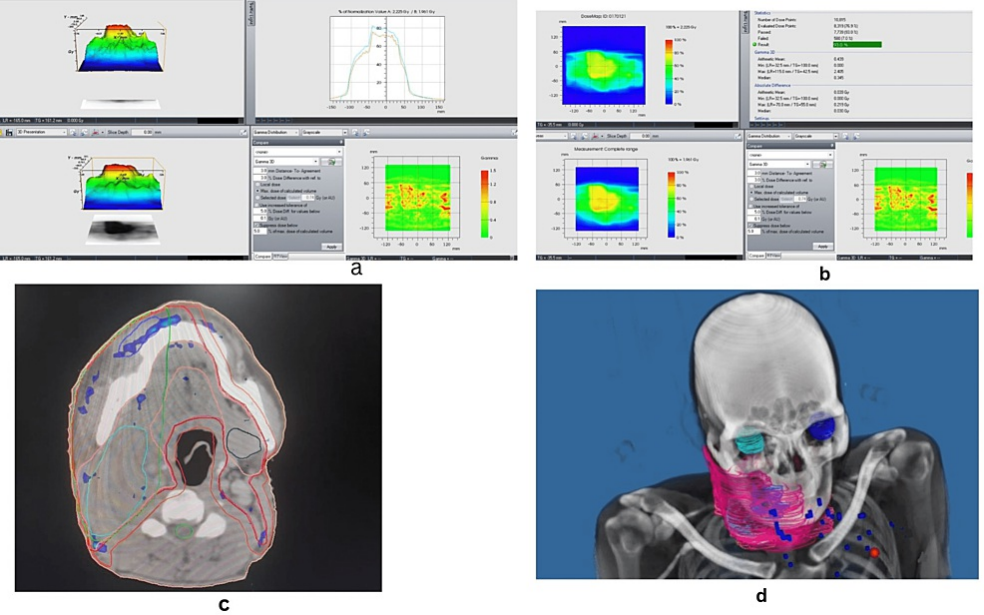
A patient diagnosed with buccal mucosa cancer. Image (a) shows a dosimetric comparison of TPS and verification plan and failing points in VeriSoft software. (b) 3D gamma index with 93% passing points with 3 mm/3% cut-off. Image (c) shows hotspots in the IMRT treatment plan (>107%). (d) CT overlay showing failing points in gamma analysis The areas of “lack of agreement” in gamma analysis should be interpreted in the context of patient anatomy for clinical decision-making, otherwise, it may potentially mask clinically relevant errors. TPS: treatment planning system; IMRT: intensity-modulated radiotherapy; 3D: three-dimensional.

Conventional IMRT QA metrics need to be considered together with the anatomical region in which dose errors are overlaid [10]. This is one of the limitations of using the IMRT QA metric if used without clinical localization of the error. The areas of “lack of agreement” in gamma analysis should be interpreted in the context of patient anatomy for clinical decision-making, otherwise, it may potentially mask clinically relevant errors [15]. This is illustrated by a case study in Figure 5. The figure illustrated the hot spots (>107% and <115% isodose areas) in the IMRT plan and the localization of “failing points” in γ analysis overlaid on CT anatomy is depicted. In a study with simultaneously integrated boost (SIB) IMRT, it was found that failed data points predominantly lie in high-dose gradient regions [16]. The “hot” or “cold” region situated over the target could have a different significance than a normal organ [17]. Software with the advantage of overlay of the failed points on CT anatomy can be advantageous for better clinical decision-making and judgment.

Recently, the integration of the γ index with radiobiological factors to obtain a better clinical perspective had been investigated [18,19]. The quantitative information on the difference between the TPS plan and the measured plan can be potentially combined with the tumor control probability (TCP)/normal tissue complication probability (NTCP) model to obtain more robust parameters with better prognostic and predictive outcomes [19]. Further studies with survival and toxicity data are needed before such parameters are used in clinical practice.

As this is a single institute study with a limited sample size, a multi-centered study involving multiple institutions, including diversified patients with variability achieving a larger sample size, may be required to generalize the clinical applicability in patient-specific quality assurance in high-precision radiotherapy.

## Conclusions

Our study shows that γ index analysis is a useful parameter for routine clinical IMRT QA. The choice of type of gamma index depends on the context of use and the degree of stringency of measurement required. 2D local and global gamma differed significantly in different directions. Average 2D and 3D global gammas were different in different anatomical regions. Average 3D global and local gammas were significantly different along directions. Local and global volumetric gammas were significantly different though correlated. No difference was observed with techniques of IMRT/VMAT. Though volumetric γ encompassed more “passing points” than 2D γ, it is recommended that failing points of γ analysis are interpreted in a clinical context keeping in mind the anatomical region where the points of failure lie. The development of a radiobiological model with γ index and survival and toxicity data can have prognostic significance and should be further investigated.
